# The rapid detection of procalcitonin in septic serum using immunoaffinity MALDI chips

**DOI:** 10.1186/s12014-023-09410-3

**Published:** 2023-05-11

**Authors:** Josef Dvorak, Jana Novakova, Lucie Kraftova, Vendula Studentova, Martin Matejovic, Jaroslav Radej, Thomas Karvunidis, Jan Horak, Marcela Kralovcova, Jaroslav Hrabak, Zuzana Kalaninova, Michael Volny, Petr Novak, Petr Pompach

**Affiliations:** 1 Department of Biochemistry, Faculty of Science, Charles University, Prague, Czech Republic; 2 Institute of Microbiology, The Czech Academy of Science, Prague, Czech Republic; 3 Institute of Biotechnology, The Czech Academy of Science, Prague, Czech Republic; 4 Biomedical Center, Faculty of Medicine in Pilsen, Charles University, Pilsen, Czech Republic; 5 Department of Internal Medicine, Faculty of Medicine in Pilsen, Pilsen University Hospital, Charles University, Pilsen, Czech Republic

**Keywords:** Procalcitonin, MALDI-TOF, Sepsis, Immunoaffinity, Ion soft landing

## Abstract

**Background:**

Sepsis is a common worldwide health condition with high mortality. It is caused by a dysregulated immune response to the pathogen. Severe infections resulting in sepsis can be also determined by monitoring several bloodstream biomarkers, one of them being pro-hormone procalcitonin (PCT). PCT concentration in the bloodstream correlates well with sepsis and in severe cases increases up to a thousand times from the healthy physiological values in a short time. In this study, we developed a rapid technique for PCT detection by MALDI-TOF mass spectrometry, that uses in-situ enrichment directly on the specialized immuno MALDI chips that are utilized as MALDI plates. The method’s ability to detect PCT was confirmed by comparing the results with LC–MS bottom-up workflow. The new method detects intact PCT by its m/z and uncovers its alternations in septic serum.

**Methods:**

The MALDI chips used for the detection of PCT were prepared by ambient ion soft landing of anti-PCT antibody on an ITO glass slide. The chips were used for the development of the rapid MALDI-TOF MS method. A parallel method based on affinity enrichment on magnetic beads followed by LC–MS/MS data-dependent peptide microsequencing was used to prove PCT presence in the sample. All samples were also tested by ELISA to determine PCT concentration prior to analyzing them by mass spectrometry methods.

**Results:**

The MALDI chip method was optimized using recombinant PCT spiked into the human serum. The PCT detection limit was 10 ng/mL. The optimized method was used to analyze 13 sera from patients suffering sepsis. The PCT results were confirmed by LC–MS/MS. The measurement of the intact PCT by the MALDI chip method revealed that sera of patients with severe sepsis have other forms of PCT present, which show post-processing of the primary sequence by cleavage of PCT, resulting in the formation of N and C termini fragments.

**Conclusions:**

Procalcitonin from human serum was successfully enriched and detected using immunoaffinity MALDI chips. The intact PCT was characterized in 13 septic patients. The method is more specific compared to non-MS-based immunoaffinity techniques and allows observation of different variants of PCT in septic patients.

**Supplementary Information:**

The online version contains supplementary material available at 10.1186/s12014-023-09410-3.

## Background

Sepsis represents a severe clinical syndrome with very high mortality and long-term morbidity of its survivors [1, 2]. It is noted that there are over 31 million cases of sepsis annually worldwide, resulting in more than 5 million deaths per year [2]. In 2016 an updated definition of sepsis was introduced to address advances made in the pathobiology, management, and epidemiology of sepsis, suggesting the need for reexamination [3]. According to the document, known as Sepsis-3, sepsis should be defined as life-threatening organ dysfunction caused by a dysregulated host response to an infection. To prevent the fatal consequences of sepsis and to properly manage the therapy, fast and accurate diagnosis is crucial [4]. There are multiple clinical diagnostic criteria that include body temperature, heart rate, systolic blood pressure, respiratory rate or altered mentation as reviewed by Singer [3]. Besides these well-established diagnostic options, another avenue to diagnose sepsis if tools of laboratory medicine are available, is offered by analytical determination of various biomarkers, one of them being PCT [5].

Procalcitonin is 116 amino acids (AA) long precursor protein of the hormone calcitonin truncated at the N-end to form 114 AA bloodstream-present form at molecular mass around 12.6 kDa [6–8]. In healthy individuals, PCT is produced by thyroid C-cells. Its normal concentration in healthy individuals is less than 0.1 ng/mL. However, the concentration might increase up to a thousand times during sepsis (hundreds of ng/mL). PCT is sensitive and highly specific for bacterial infections, possessing rapid kinetics usually reflecting response to antibiotic therapy. These facts make PCT a useful biomarker for clinical diagnostics of sepsis [9, 10] with known advantages over other blood-based markers (e.g. C-reactive protein, or white blood cells count). Once the PCT level is at a maximum value, it doesn’t change unless the patient's condition improves after successful treatment. Therefore PCT isn’t only a valuable biomarker of sepsis and septic shock, but it can also be used as an indicator of treatment effect [11].

All currently FDA-approved detection methods are immunoassays that use anti-PCT antibodies to determine PCT concentration, e.g. ELISA [12]. Although these methods are sensitive, they may suffer from false positivity caused by antibody cross-reactivity. Immunoaffinity approaches coupled with mass spectrometry overcome the problem with false positivity by detecting precise molecular weight and identifying the exact forms of the molecular target, such as PCT. On the other hand, MS-based techniques are usually time-consuming, less robust, and can have problems to achieve ELISA limits of detection.

Here, we present a new method that combines in-situ enrichment of PCT on antibody-functionalized MALDI chips with MALDI-TOF MS detection.

The affinity surfaces used as MALDI chips were prepared by ambient ion soft landing of PCT antibody. This technology, previously well-described [13–15], allows modification of surfaces with a wide variety of ions. In ambient ion soft-landing ions are generated by soft electrospray ionization at atmospheric pressure, desolvated in a heated tube, and landed on a conductive surface such as MALDI plates. This approach allows effective and unique modification of surfaces without dramatical loss of ions while keeping their biological activity [16]. Several applications of MALDI surfaces prepared by ambient ion soft-landing for life science and clinical diagnostics have been recently presented [15, 17–21]. Detection of PCT represents a challenging task because of its low abundance in human serum and throughput requirements of clinical assays. The workflow described in the presented work uses detection from serum by in-situ enrichment on MALDI chips modified with commercially available anti-PCT antibody. The lab-on-plate sample preparation workflow is followed by standard MALDI-TOF MS detection. The assay was optimized by using recombinant PCT and successfully used to analyze samples of 13 septic patients with PCT

## Methods

### Chemicals and materials

Glass slides coated with a thin layer of indium-tin-oxide (ITO) were purchased from Bruker Daltonics (Massachusetts, USA). Recombinant PCT was purchased from BioVendor (Brno, Czech Republic) and an anti-PCT antibody from Fitzgerald (Massachusetts, USA). Magnetic beads Dynabeads^™^ Co-Immunoprecipitation Kit was purchased from Thermo Fisher Scientific (Massachusetts, USA). All other chemicals were obtained from Merck (Darmstadt, Germany).

### Manufacturing of immunoaffinity MALDI ITO chips

Immunoaffinity chips were prepared using a previously described lab-built apparatus for an ambient ion-soft landing [22]. The ambient ion-soft landing technique was used to immobilize the anti-PCT antibody on standard ITO-coated glass slides (Bruker Daltonics, USA). The geometry of 16 spots array per chip was defined by an adhesive foil mask with a 2 mm spot diameter and 9.0 mm center-to-center distance. Anti-PCT antibody was sprayed from 0.6 mg/mL solution of 50 mM ammonium bicarbonate buffer (AMBIC), pH 7.8. The solution was delivered by the syringe pump at a flow rate of 60 μL/h through the 20-μm capillary connected to the microspray emitter. 1.5 kV of positive voltage was applied to the syringe needle to create an electrospray. Nebulization was further supported by the stream of nitrogen gas preheated to 40–50 °C. The newly prepared chip was washed in water after soft landing to remove any excess material to only allow antibody bound to the surface to stay on the chip.

### Optimizing the PCT detection method

#### In-situ enrichment and detection of recombinant PCT

Recombinant human PCT (BioVendor, Czech Republic) was used as a standard for optimization of in-situ enrichment and MS detection. Procalcitonin was diluted in human serum at concentrations of 42 µg/mL, 14 µg/mL, 4.7 µg/mL, 1.6 µg/mL and 0.5 µg/mL. One microliter of the sample was loaded onto the spot of landed antibody on the ITO chip and incubated for 1 h at room temperature inside the Petri dish to minimalize evaporation of the sample. After incubation, the chip was washed three times for 5 min in PBS buffer and 1 min in deionized water and allowed to dry at room temperature. MALDI matrix (2,5-dihydroxyacetophenone, DHAP) was prepared according to a previously published protocol [21]. 0.6 μL of DHAP solution was pipetted on the chip and gently mixed several times by the pipette tip to obtain homogenous crystals.

Chips with enriched analyte and deposited MALDI matrix were placed into the MALDI slide adapter II holder (Bruker Daltonics) and aspirated into the MALDI-TOF mass spectrometer (Autoflex Speed, Bruker Daltonics). Procalcitonin was analyzed in a positive linear mode in the mass range 4 400–16 800 m/z. The laser frequency was set to 1 kHz, and 20 000 shots were accumulated for each sample using the partial random walk function. Each sample was in-situ enriched and measured at three different positions. In a different set of experiments, PCT was also analyzed by MALDI FT-ICR mass spectrometer (15 T solariX XR, Bruker Daltonics) in the mass range 800–16 000 m/z. Twenty-four spectra were accumulated with data acquisition of 2 M. MALDI-TOF MS data were processed by FlexAnalysis 3.0 and MALDI-FT-ICR data were processed by DataAnalysis 5.0 software (Bruker Daltonics).

#### Procalcitonin sample treatment

To improve the detection limit of the method, recombinant procalcitonin spiked in human serum (100 μL) was diluted with LC–MS water in the 1:2 ratio and vortexed for 10 s. The sample was further diluted by acetonitrile in the 1:4.5 ratio, vortexed, and sonicated for 10 min in an ultrasonic bath with ice. After centrifugation at 8000 ×*g* for 15 min at 4 °C, the supernatant containing PCT was lyophilized using a SpeedVac concentrator.

### Procalcitonin detection in patient's serum

#### Patient samples

Samples of human venous blood (5 mL) were collected using the VACUETTE blood-collection system (Greiner Bio-One) containing 5 mM EDTA. Serum was separated by 10 min centrifugation at 1 700 ×*g* and immediately frozen to − 80 °C. The concentration of PCT in each sample was determined by ELISA. Each serum sample was then split into two aliquots and further analyzed either by magnetic beads or by functionalized MALDI chips.

#### In-situ enrichment of PCT using functionalized MALDI chips

The patient's sera were analyzed similarly to the recombinant PCT: 100 μL of serum was mixed with 200 μL of water and 450 μL of acetonitrile. Further processing was identical as described before for recombinant PCT samples. Samples were deposited in triplicates on the functionalized spots on the MALDI chip and analyzed by MALDI-TOF and MALDI FT-ICR mass spectrometers. Both mass spectrometers were externally calibrated using myoglobin and cytochrome C.

The limit of detection and the dynamic range were determined by measuring recombinant PCT spiked into a human serum at concentrations 1000 ng/mL, 500 ng/mL, 100 ng/mL, 50 ng/mL, 10 ng/mL and 5 ng/mL. A 6 × 100 µL of each sample was concentrated by acetonitrile enriched on the MALDI plate and analyzed using MALDI-TOF and MALDI FT-ICR. For determination of the coefficient of variation, the recombinant PCT was spiked into a human serum pool at a concentration of 140 ng/mL and split into 16 × 100 µL fractions, and treated and analyzed as described above.

#### Enrichment of PCT using magnetic beads

Magnetic beads (Dynabeads^™^ Co-Immunoprecipitation Kit, Thermo Fisher Scientific) were prepared according to the manufacturer's instructions, the brief summary of the protocol follows. The PCT antibody used for modification of magnetic beads was the same as the PCT antibody used for the functionalization of the MALDI chips. One milliliter of C1 solution (included in the Dynabeads kit) was used to rinse 7.5 mg of beads and the rinsing solution was separated in the magnetic rack. In the next step, 375 μL of 0.15 mg/mL anti-PCT antibody in C1 solution was added and mixed with the beads, followed by the addition of 375 μL of C2 solution. The beads were incubated overnight on a rotator at 4 °C and then rinsed as follows: 1 × 800 µL HB solution, 1 × 800 µL LB solution, 2 × 800 µL SB solution and again with 1 × 800 µL of SB solution for 15 min on rotator at laboratory condition with suspense transfer to a new microtube, 4 × 800 µL 1 × IP solution containing 150 mM NaCl and Halt^™^ Protease and Phosphatase inhibitor cocktail (Thermo Scientific). The magnetic beads with coupled antibody were stored in 700 µL SB solution with 0.02% NaN_3_.

Before the use, magnetic beads were rinsed 3 × in 800 µL of PBS with 0.1% BSA. The patient serum was incubated with the beads for 45–60 min on a rotator at 4 °C. After immunoprecipitation, the liquid phase was removed using a magnetic rack and a pipette. The beads were rinsed 3 × in 800 µL of PBS with 0.1% BSA and then washed 1 × in 800 µL of LWB solution for 5 min using a rotator at laboratory conditions. Beads transferred to a new microtube were washed 3 × with 800 µL of LWB solution.

Proteins were eluted from the beads by the addition of 2 × 300 µL of elution buffer (0.5 mM EDTA, 0.3 mM NH_4_OH in LC–MS water). Samples were dried by SpeedVac concentrator and resuspended in 20 µL of 5% acetic acid in LC–MS water with 10 mM dithiothreitol (DTT). For intact mass spectrometry, two microliters of sample were spotted onto the MALDI steel plate and covered with 0.6 µL of DHAP matrix.

#### Bottom-up detection of PCT enriched by magnetic beads

For LC–MS/MS analysis, five microliters of sample were mixed with 20 µL of 50 mM ammonium bicarbonate. The sample pH was adjusted with NH_4_OH solution to pH 7–8. The sample was incubated at 60 °C for 40 min. After cooling down to room temperature, iodoacetamide was added to the final concentration of 30 mM, and the sample was incubated for 30 min in the dark. Additional DTT was added to the final concentration of 50 mM to stop the alkylation reaction. Trypsin (0.1 µg/mL) was added, and the reaction mixture was incubated overnight at 37 °C.

Samples were analyzed using a liquid chromatography system Agilent 1200 (Agilent Technologies) connected to the timsToF Pro PASEF mass spectrometer equipped with Captive spray (Bruker Daltonics). The mass spectrometer was operated in a positive data-dependent mode. Five microliters of peptide mixture were injected by autosampler on the C18 trap column (UHPLC Fully Porous Polar C18 2.1 mm ID, Phenomenex). After 5 min of trapping at a flow rate of 20 μL/min, peptides were eluted from the trap column and separated on a C18 column (Luna Omega 3 μm Polar C18 100 Å, 150 × 0.3 mm, Phenomenex) by a linear 35 min water − acetonitrile gradient from 5% (v/v) to 35% (v/v) acetonitrile at a flow rate of 4 μL/min. The trap and analytical columns were both heated to 50 °C. Parameters from the standard proteomics PASEF method were used to set timsTOF Pro. The target intensity per individual PASEF precursor was set to 6000, and the intensity threshold was set to 1500. The scan range was set between 0.6 and 1.6 V s/cm^2^ with a ramp time of 100 ms. The number of PASEF MS/MS scans was 10. Precursor ions in the m/z range between 100 and 1700 with charge states ≥ 2 + and ≤ 6 + were selected for fragmentation. The active exclusion was enabled for 0.4 min.

The raw data were processed by PeaksStudio 10.0 software (Bioinformatics Solutions, Canada). The search parameters were set as follows: enzyme–trypsin (specific), carbamidomethylation as a fixed modification, oxidation of methionine, and acetylation of protein N-terminus as variable modifications. Database: UniProt (human, virus and bacteria, 01/2021). The mass spectrometry data have been deposited to the ProteomeXchange Consortium via the PRIDE partner repository with the dataset identifier PXD035456 [23].

## Results

**Fig. 1:**
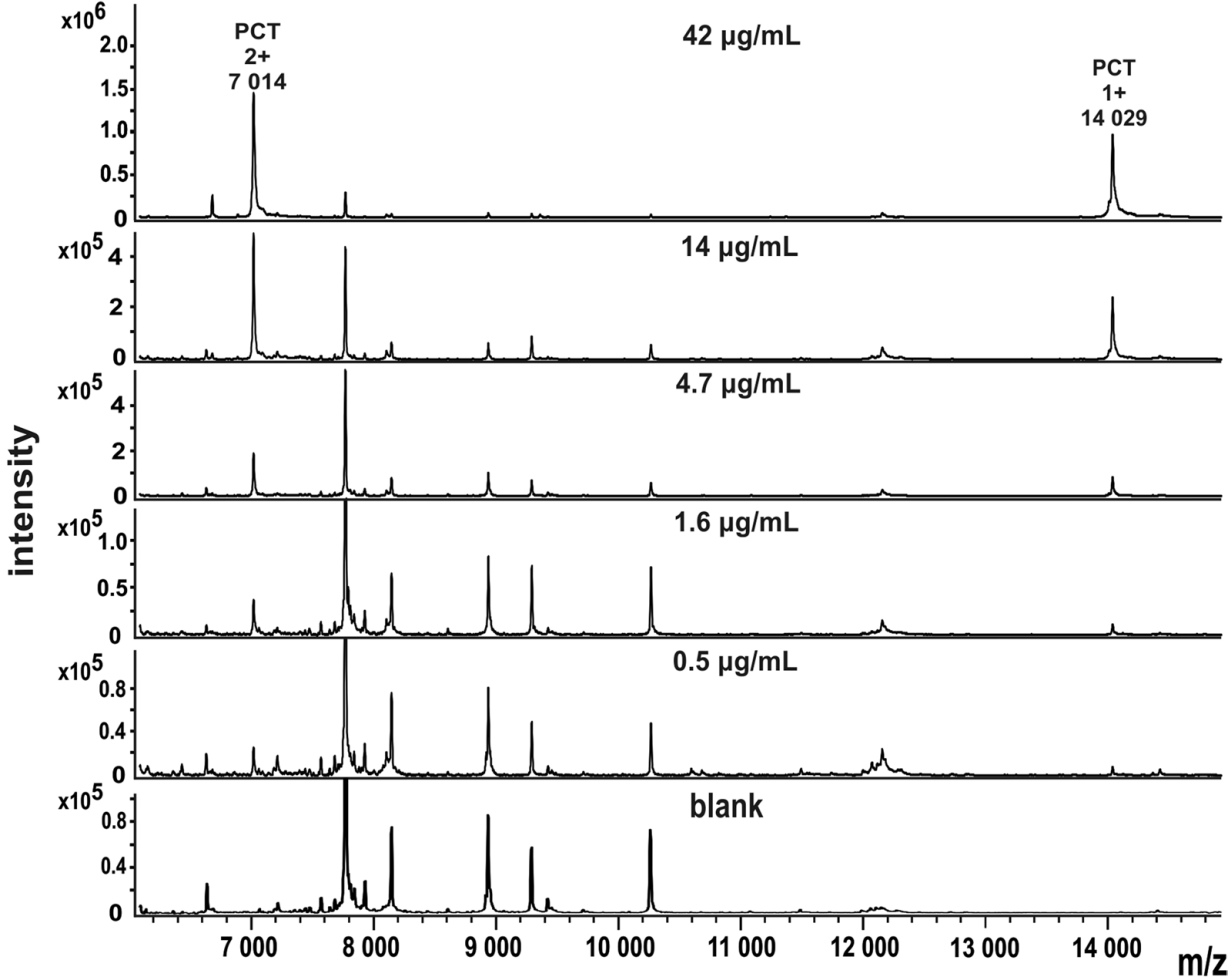
MALDI-TOF MS spectra of recombinant PCT spiked in human serum at different concentrations enriched and analyzed by functionalized MALDI chips. The bottom panel shows spectrum of serum without spiked PCT (blank)

The MALDI chips modified by the ambient landing of the human PCT antibody were first evaluated by analyzing samples of human recombinant PCT spiked into the human pooled serum of healthy individuals at different concentrations. The ions corresponding to singly and doubly charged recombinant PCT were observed at m/z 14 029 and 7 014 (Fig. 1). The doubly charged ions show better peak resolution and signal intensity. That is why DHAP was a preferred MALDI matrix over sinapic acid, because it supported their formation [21]. Figure 1 shows analysis of serum samples with spiked PCT at different concentration using the MALDI chips prepared by soft landing of the anti-PCT antibody. The signal-to-noise ratio of doubly charged ion of PCT at the lowest detected concentration (0.5 μg/mL) was 12.

To improve the limit of detection, serum samples with recombinant PCT were concentrated by acetonitrile precipitation of a major amount of serum proteins to leave the PCT in a supernatant. Two different approaches for concentration using acetonitrile were tested. The efficiency of the concentration method was confirmed by immunostaining using a PCT antibody. The best results were achieved when acetonitrile was added to the diluted serum at a 1:4.5 (v/v) ratio without addition of acid (Additional file 1: Fig. S1).

**Fig. 2:**
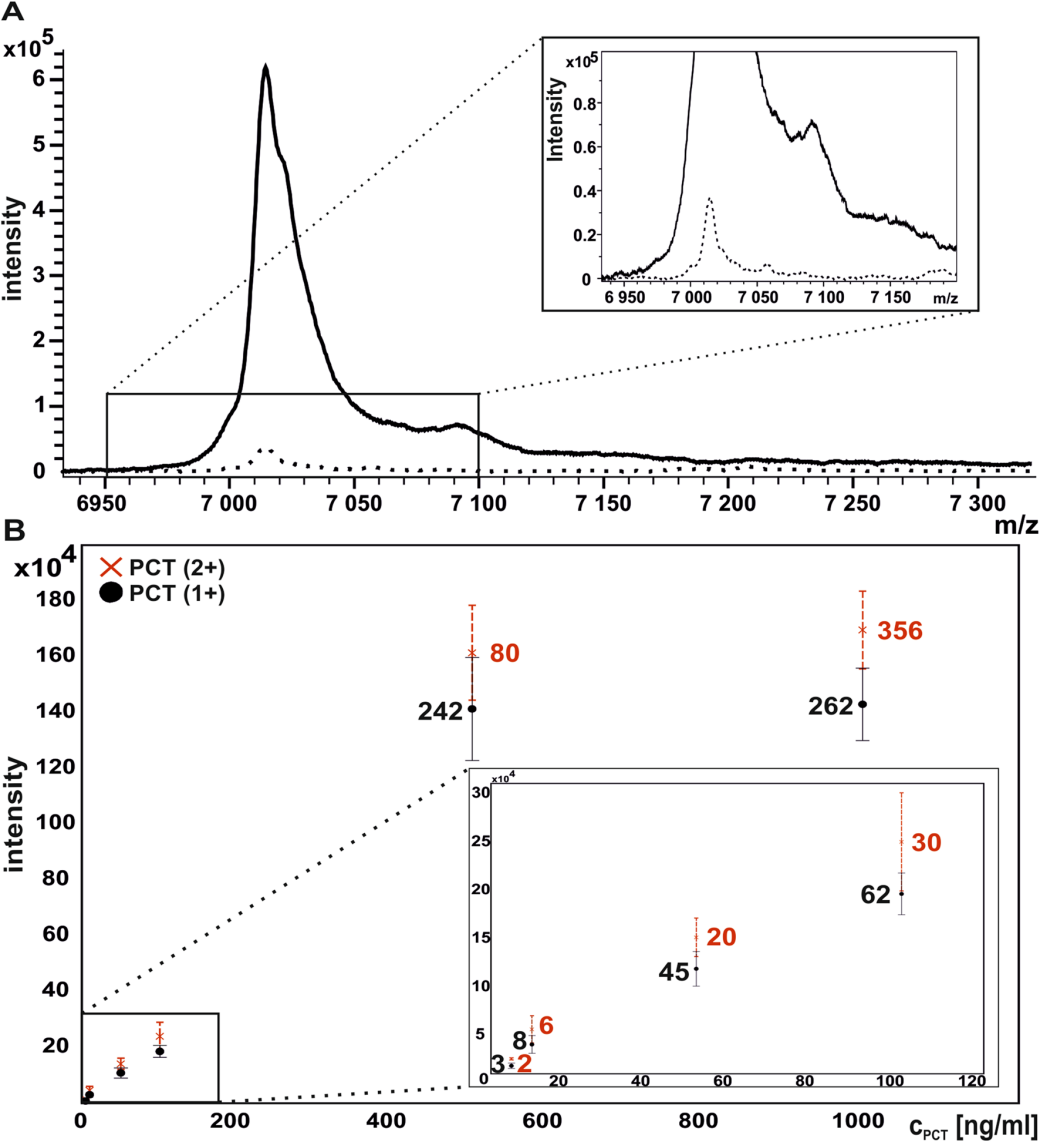
**A** MALDI-TOF MS spectra of in-situ enriched recombinant PCT (140 ng/mL) concentrated by acetonitrile (solid line) and without concentration treatment (dashed line). **B** Relative intensities of singly and doubly charged ions at different concentrations of recombinant PCT. The PCT concentrations are 5; 10; 50; 100; 500; 1000 ng/mL The numbers represent S/N ratios for singly (black) and doubly (red) charged ions

The concentration procedure increased the sensitivity of the method by the factor of 15 calculated based on the comparison of ion signal intensities (Fig. 2A). The limit of detection was improved to 10 ng/mL (Additional file 2: Fig. S2). Highly concentrated samples (500 ng/mL and 1000 ng/mL) were out of the linear dynamic range of the method (Fig. 2B).

The coefficient of variation (CV) of the PCT in serum was determined using 16 samples with spiked recombinant PCT at a concentration of 140 ng/mL that were concentrated using the acetonitrile treatment. This concentration level is within the linear detection range and simulates PCT blood concentration during severe sepsis. The CV value was 25% for singly and 22% for doubly charged PCT ions. The chip-to-chip CV was 14% for singly charge ion and 10% for doubly charge ions.

### Detection of PCT in patient's samples

The developed technique was tested for the detection of PCT in patient samples. The presence of PCT was first determined by using the ELISA method. Thirteen samples of septic patients with PCT concentrations above 100 ng/mL were selected for evaluation of MALDI chips. Each patient's serum was divided into two aliquots. One was used for enrichment on magnetic beads with LC–MS detection and the other for enrichment on functionalized MALDI chips with MALDI-TOF MS detection. Samples for MALDI chip enrichment were processed by the acetonitrile concentration procedure. The physiological human PCT is different from the recombinant protein used for optimization: the primary sequence does not include the Histidine tag anchor and the 2 N-terminus amino acids are cleaved. That is why the physiologically occurring PCT has a different molecular mass and it is detected by MALDI-MS at different m/z values (12626 for singly charged and 6308 for doubly charged ions) than the recombinant version. To confirm that the accurate mass of PCT corresponds to the expected sequence, each sample was also analyzed by a high-resolution accurate mass MALDI FT-ICR mass spectrometer (Additional file 3: Fig. S3). It confirmed the presence of PCT by detection of the doubly charged ion at m/z 6 310.32, but a singly charged ion was not observed.

**Fig. 3:**
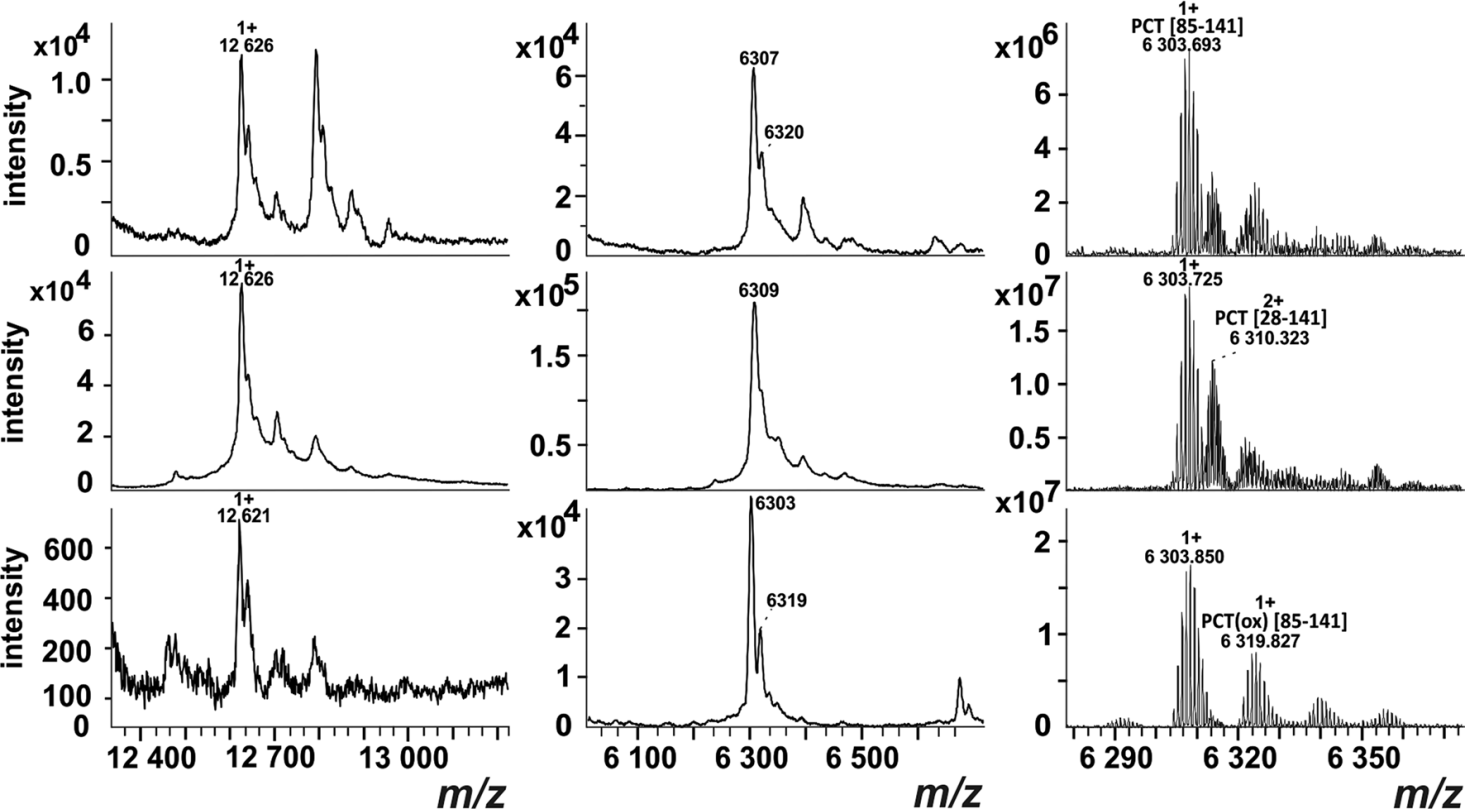
Detection of PCT using the MALDI chips in three selected septic patients (from top to bottom). The figure shows detailed spectra of singly and doubly charged ions of PCT and its cleaved forms measured by MALDI-TOF MS (left and center) and by MALDI FT-ICR (right). In the third patient's sample, the intensity of the doubly and singly charged ions of PCT is very low compared to the intensity of singly charged fragment ion at m/z 6 303.850, which indicates that most of the PCT was converted into the fragment form

Interestingly, analysis of PCT in patient samples also revealed the presence of other forms of PCT as shown in Fig. 3. A dominant singly charged ion at m/z 6303.85 and its oxidized form at m/z 6319.83 were detected. This ion was interpreted as a PCT fragment C^85^GNLSTCMLGTYTQDFNKFHTFPQTAIGVGAPGKKRDMSSDLERDHRPHVSMPQNAN^141^ with cysteines forming a disulfide bond. A doubly charged ion at m/z 6310.32, which corresponds to noncleaved PCT, was still present in most samples, but the modified forms were dominant. Figure 3 shows an example of three selected patient samples. All PCT MALDI-TOF MS spectra from septic patients’ sera samples are shown in the Additional file 3: Figure S3.

**Fig. 4:**
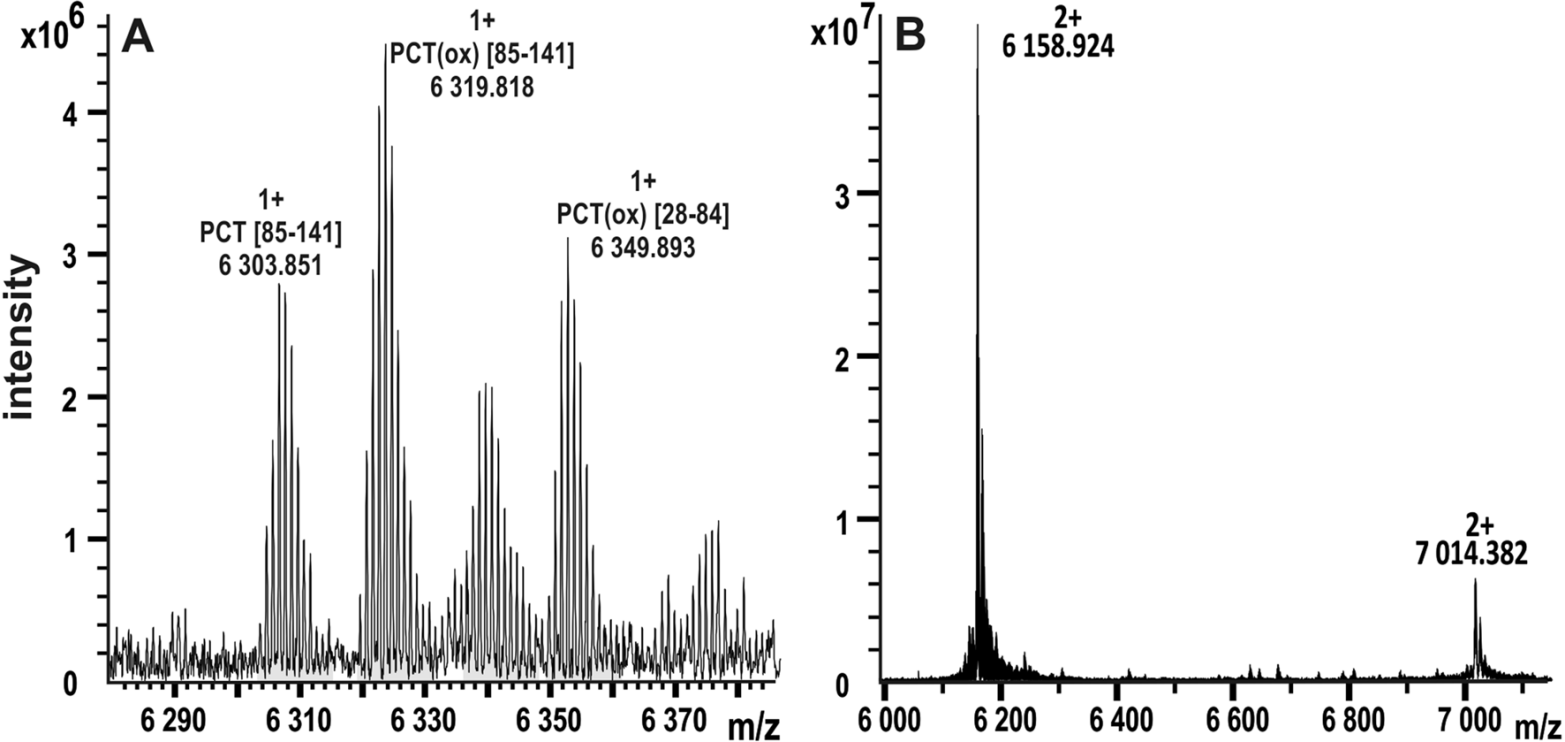
**A** Detailed MALDI FT-ICR spectrum of PCT from septic patient enriched by MALDI chip. Fragments of PCT and their oxidized forms corresponding to cleavage at the R84-C85 site were detected. **B** MALDI FT-ICR spectrum of human recombinant PCT spiked in serum and incubated at 37 °C shows no equivalent fragments. This indicates that the origin of the fragments is not related to sample degradation

Another fragment ion at *m/z* 6349, (1 +) that was observed in the patient’s serum was assigned as a PCT fragment:

F^28^RSALESSPADPATLSEDEARLLLAALVQDYVQMKASELEQEQEREGSSLDSPRSKR^84^ (Fig. 4A). To investigate if the fragments originate simply from sample degradation, human pooled serum spiked with recombinant PCT was incubated for 3 h at 37 °C. The serum was processed by acetonitrile concentration procedure and PCT was detected using MALDI chips. The recombinant PCT was truncated at its N-terminus, but PCT cleavage fragments that are present in the patient samples (Fig. 4A) were not observed in the incubated sample (Fig. 4B).

The second aliquot was enriched on anti-PCT antibody-modified beads. Interestingly, samples that were enriched using magnetic beads, eluted, and analyzed by MALDI-TOF MS showed more complex spectra in comparison with samples enriched and analyzed directly on MALDI chips (Additional file 4: Fig. S4). The aliquot eluted from the beads was also analyzed by a bottom-up LC–MS/MS workflow. The qualitative results confirmed PCT in all 13 samples in accordance with the analysis on MALDI chips. (Because the bottom-up results were searched against a microbiology proteome database as well, it was identified that one of the patient samples contained the RTX-I toxin from *Actinobacillus pleuropneumoniae*. The RTX-I toxin was matched by 41 peptides with an FDR value < 1% covering 38% of the protein sequence (Additional file 5: Fig. S5). The PCT enriched from the serum with RTX-I toxin was fully converted into its cleaved forms. The full list of proteins identified in the patient's samples analyzed by LC–MS/MS is available in (Additional file 6: Table S1).

## Discussion

The purpose of this work was to develop a mass spectrometry technique for the detection and characterization of human PCT that would be compatible with the requirement for clinical management of the sepsis. MALDI-TOF MS was chosen as a technique that is relatively simple, provides rapid results, and is common in clinical microbiology laboratories and has been used in clinical chemistry as well [24]. The specific advantage of our approach is that the entire workflow can be performed in-situ on the MALDI chip and detected by any standard MALDI-TOF mass spectrometer, including standard microbiology biotypers.

MALDI chips used in the current work were prepared by ambient ion soft-landing of antibodies. This technique was previously reported as a platform for detecting proteins in complex matrices. Several applications of MALDI chips and lab on plate concept have already been presented, including protein digestion by different proteases, glycopeptide enrichment by immobilized lectins, haptoglobin phenotyping, or quantification of human carbohydrate-deficient transferrin using immobilized antibodies [15, 19, 21, 25, 26]. The current study demonstrates a possibility to immobilize antibodies by ambient ion soft landing with retention of their immunoaffinity and use them effectively for the immunoMALDI workflow. This workflow allows effective detection of analytes in relatively complex clinical assays, as described before [27].

We utilized the acetonitrile concentration procedure, which precipitated major amount of serum proteins, while leaving PCT in the acetonitrile phase, to reduce the complexity of the sample matrix and to improve the limit of detection. The acetonitrile procedure in combination with MALDI chips allowed the detection the recombinant PCT by MALDI-TOF MS at a concentration level of 10 ng/mL. This is not a sufficient limit of detection for monitoring PCT in healthy individuals (0.1 ng/mL or less), nor in patients with mild infections (2 ng/mL) [28], but can be used for the detection of high levels of PCT that are present in the serum during severe sepsis.

Procalcitonin in a similarly small cohort of septic patients (PCT concentration > 100 ng/mL) was analyzed in a 2001 study by Weglohner et al. [8]. The results showed that two amino acids from the N-terminus are missing. This is in agreement with our results that also show the N-terminal truncated form of PCT. No N-glycosylation of PCT was found in our results, although the primary amino acid sequence of PCT contains N-glycosylation motive NLS. This is again consistent with results obtained by Weglohner [8].

More recently, a complex bottom-up LC–MS/MS workflow that includes two solid phase extraction steps was published [29]. The bottom-up LC–MS/MS approach allowed to achieve sub ng/mL limits of detection, but it is elaborate and provides quantification on the peptide level, without information about different PCT variants in the sample. The presented method based on MALDI chips and intact PCT detection is less sensitive, but the measurement of intact protein provides information about the different forms of primary structure in septic patients. The mass spectrum of human PCT analyzed by MALDI chips with MALDI-TOF MS detection contained singly and doubly charged ions of PCT, corresponding to its primary amino acid sequence. Oxidation of PCT, in the form of the addition of 16 Da was also present. The PCT fragments were identified by high-resolution accurate mass MALDI FT-ICR and appeared in the spectra of all septic patients. It seems that for severe septic states, the PCT exists as a mixture of intact and cleaved forms. The cleavage of PCT in sera from septic patients might be caused by the increased presence of proteases during sepsis. To exclude possible PCT degradation during sample processing, recombinant PCT was incubated in healthy individual serum at 37 °C for 3 h, processed as described before, and detected by MALDI-TOF MS and MALDI FT-ICR MS. It was shown that incubation under physiological conditions and follow-up sample processing, including acetonitrile concentration, did not cause cleavage of PCT into two observed forms. Only a partial truncation of the PCT N-terminus was observed. As mentioned above, detailed information about PCT fragments can not be obtained by ELISA methods that are not specific enough or by LC–MS/MS bottom-up approaches that only detect PCT on the level of trypsinated peptides. The MALDI chip technique that detects all intact forms of PCT represents not only specific and rapid approach, but also offers rich information about the biochemical transformation of PCT in severe sepsis. Interestingly, it was identified by LC–MS/MS that one patient sample contained RTX-I, a toxin from *Actinobacillus pleuropneumoniae.* Using classical bacteriological examination (i.e., cultivation of blood and bronchoalveolar lavage), however, we detected no significant pathogen including *A. pleuropneumoniae* (data not shown). The identified RTX-I toxin may indicate the presence of a bacterium in the bloodstream. Furthermore, the serum of this patient contained no intact PCT, but only its cleaved forms. This could indicate that sepsis severity is reflected in the ratio of intact and cleaved PCT forms. To monitor this parameter, only intact mass spectrometry methods can be utilized, because ELISA is not specific enough and bottom-up approaches detect the PCT on the level of digested peptides.

## Conclusions

The in-situ enrichment and detection of PCT from human serum using functionalized immunoMALDI chips have been reported in this study. The MALDI-compatible chips were prepared by ambient ion soft landing of PCT antibody on ITO glass slides. The method was optimized using human serum spiked with recombinant PCT protein and then used on patient serum samples. The limit of detection was estimated to be 10 ng/mL. The MALDI chip technique is capable of detecting and distinguishing different forms of PCT. It was observed that PCT of patients suffering severe sepsis undergoes cleavage and exists in several forms. The advantage of MALDI chip technology is in rapid detection and the ability to describe PCT variants, including truncated and modified forms.

## Supplementary Information

**Additional file 1: Figure S1**. Monitoring of concentration efficiency by immunostaining. **M**—protein standard; **1—**supernatant after acidic concentration**; 2—**pelet after acidic concentration**; 3—**serum with recombinant PCT**; 4—**supernatant after acetonitrile concentration**; 5—**pelet obtained after acetontril concentration.

**Additional file 2: Figure S2**. MALDI-TOF spectrum of in-situ enriched intact recombinant PCT spiked in human serum at LOD concentration 10 ng/mL.

**Additional file 3: Figure S3**. In-situ detection of PCT in 13 septic patients. Detailed spectra of singly and doubly charged ions of PCT measured by MALDI-TOF (left and center) and detailed spectra of PCT measured by MALDI FT-ICR (right).

**Additional file 4: Figure S4**. MALDI-TOF of intact PCT from septic patient`s serum using MALDI chips (**A**) and using immunoaffinity magnetic beads (**B**).

**Additional file 5: Figure S5**. Sequence coverage of Calcitonin and RTX-I toxin. The blue lines represent identified peptides by LC-MS/MS

**Additional file 6: Table S1**. The list of proteins identified in the patient's samples analyzed by LC–MS/MS.

## Data Availability

The LC–MS/MS data have been deposited to the ProteomeXchange Consortium via the PRIDE partner repository with the dataset identifier PXD035456.

## Abbreviations

AA: Amino acids
AMBIC: Ammonium bicarbonate
DHAP: 2,5-Dihydroxyacetophenone
DTT: Dithiothreitol
EDTA: Ethylenediaminetetraacetic acid
ELISA: Enzyme-linked immunosorbent assay
FDR: False discovery rate
FT-ICR: Fourier-transform ion cyclotron resonance
ITO: Indium-tin-oxide
LC–MS: Liquid chromatography mass spectrometry
MALDI: Matrix-assisted laser desorption/ionization
MS: Mass spectrometry
PASEF: Parallel accumulation and serial fragmentation
PBS: Phosphate-buffered saline
PCT: Procalcitonin
PRIDE: Proteomics identifications database
TOF: Time of flight
