# Supplementary figures and images for: The rapid detection of procalcitonin in septic serum using immunoaffinity MALDI chips
Source: Clin Proteomics. 2023 May 11;20:20. doi: 10.1186/s12014-023-09410-3 (PMC10176672; doi:10.1186/s12014-023-09410-3)

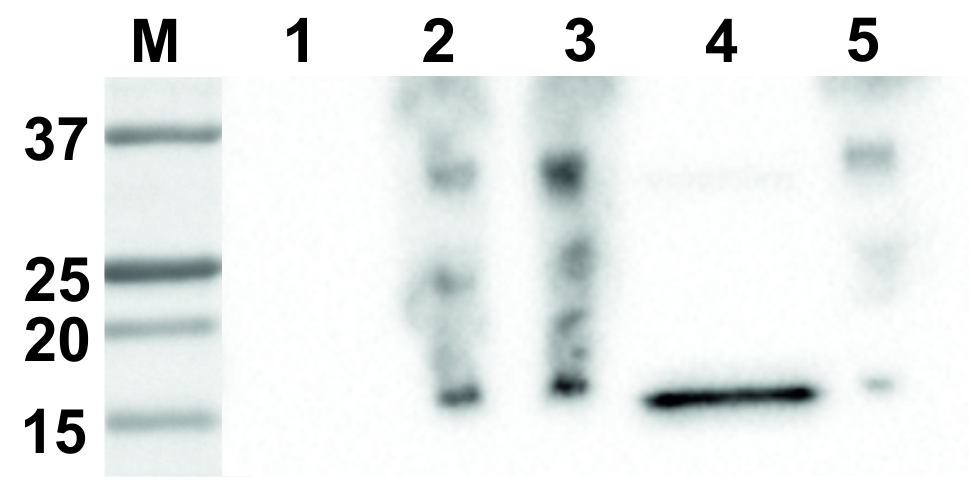

Supplement: Supplementary file 1 — Additional file 1: Figure S1. Monitoring of concentration efficiency by immunostaining. M—protein standard; 1—supernatant after acidic concentration; 2—pelet after acidic concentration; 3—serum with recombinant PCT; 4—supernatant after acetonitrile concentration; 5—pelet obtained after acetontril concentration. [file 12014_2023_9410_MOESM1_ESM.tif]

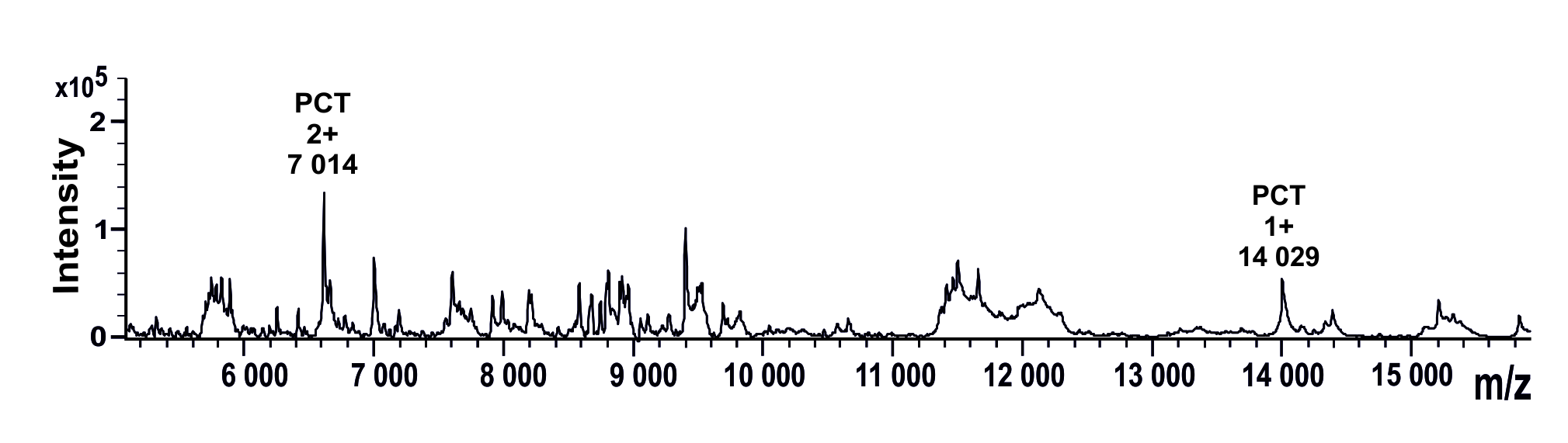

Supplement: Supplementary file 2 — Additional file 2: Figure S2. MALDI-TOF spectrum of in-situ enriched intact recombinant PCT spiked in human serum at LOD concentration 10 ng/mL. [file 12014_2023_9410_MOESM2_ESM.tif]

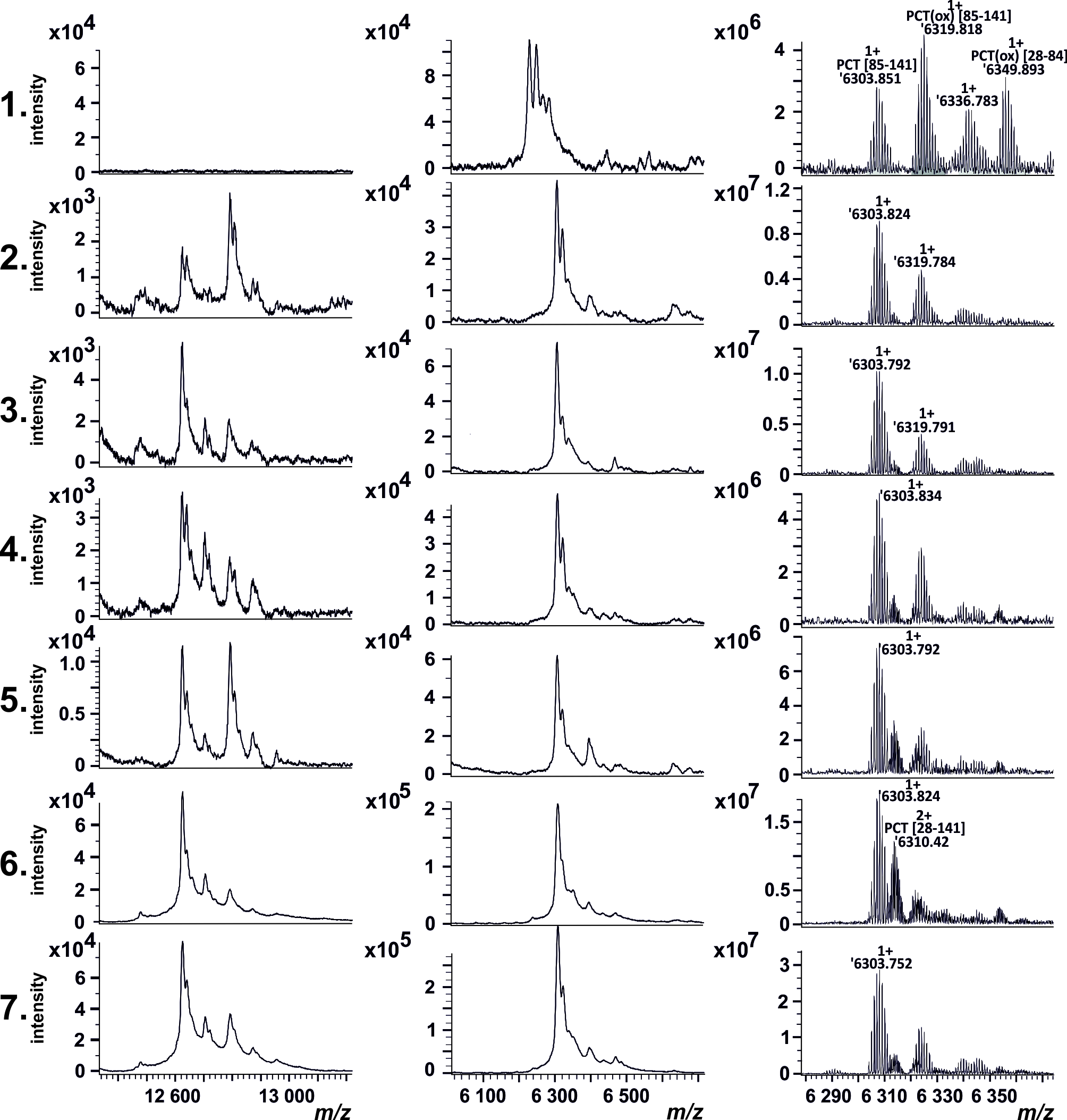

Supplement: Supplementary file 3 — Additional file 3: Figure S3. In-situ detection of PCT in 13 septic patients. Detailed spectra of singly and doubly charged ions of PCT measured by MALDI-TOF (left and center) and detailed spectra of PCT measured by MALDI FT-ICR (right). [file 12014_2023_9410_MOESM3_ESM.zip › Fig. S2/Fig. S2A_revised.tif]

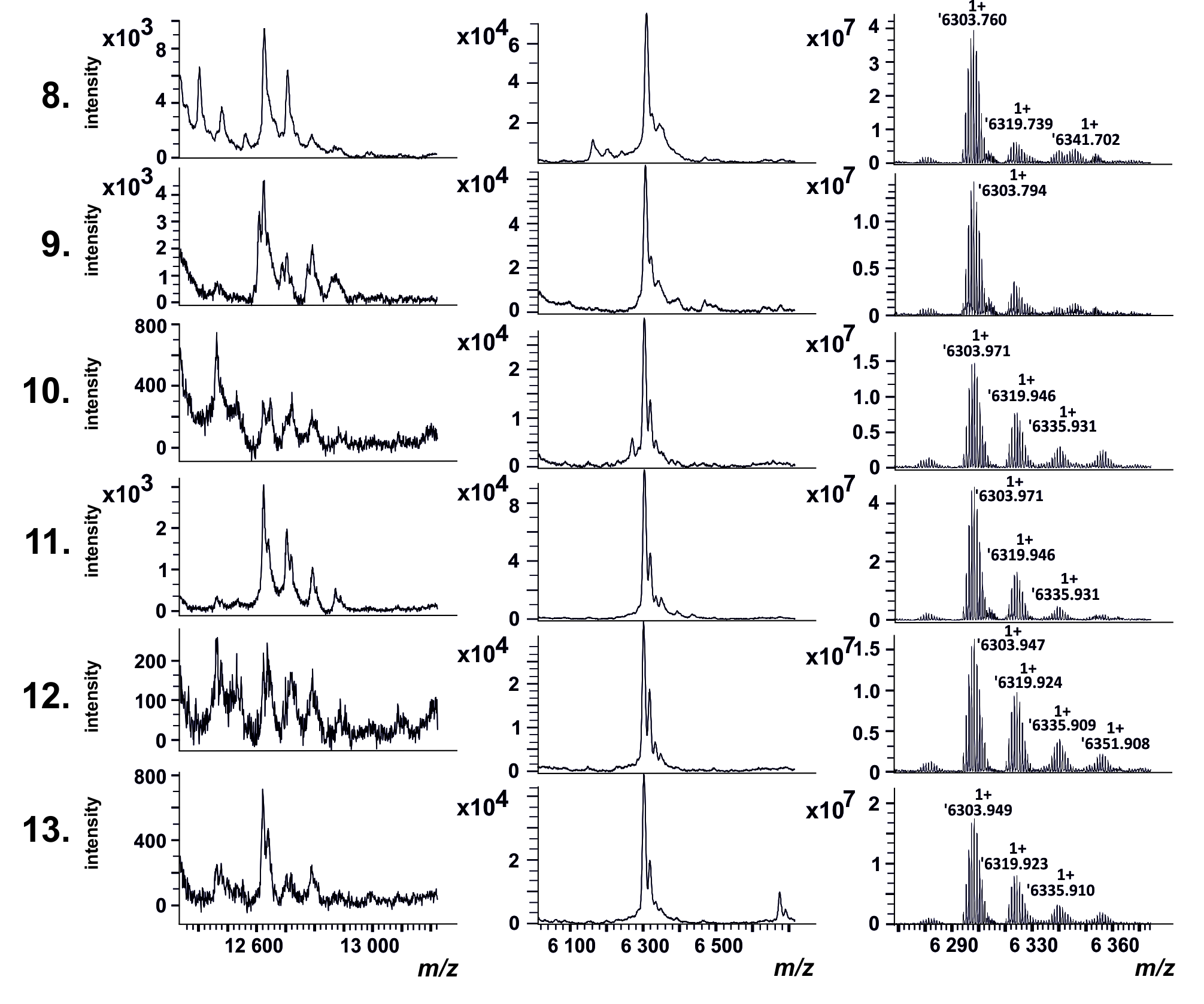

Supplement: Supplementary file 3 — Additional file 3: Figure S3. In-situ detection of PCT in 13 septic patients. Detailed spectra of singly and doubly charged ions of PCT measured by MALDI-TOF (left and center) and detailed spectra of PCT measured by MALDI FT-ICR (right). [file 12014_2023_9410_MOESM3_ESM.zip › Fig. S2/Fig. S2B_revised.tif]

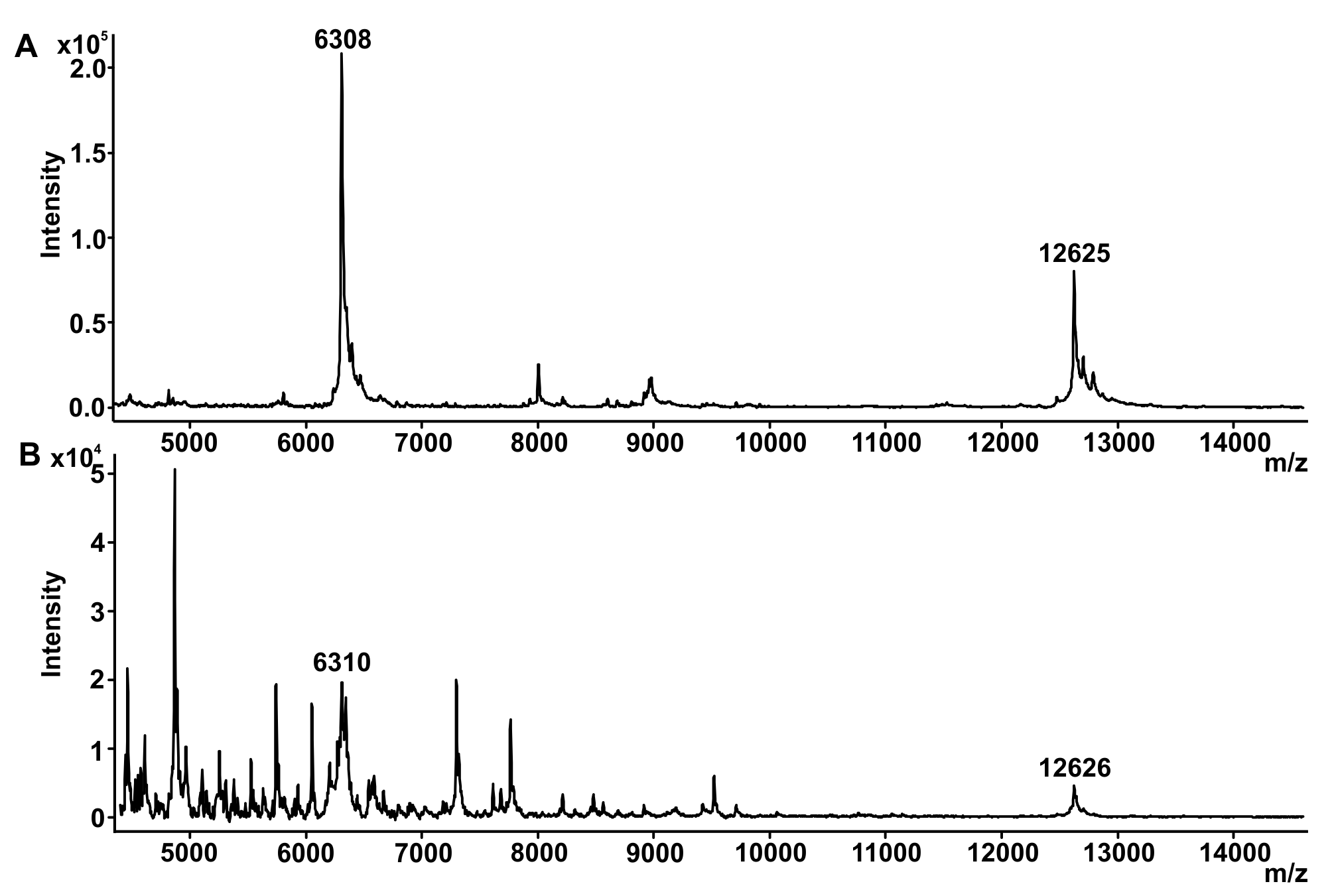

Supplement: Supplementary file 4 — Additional file 4: Figure S4. MALDI-TOF of intact PCT from septic patient`s serum using MALDI chips (A) and using immunoaffinity magnetic beads (B). [file 12014_2023_9410_MOESM4_ESM.tif]

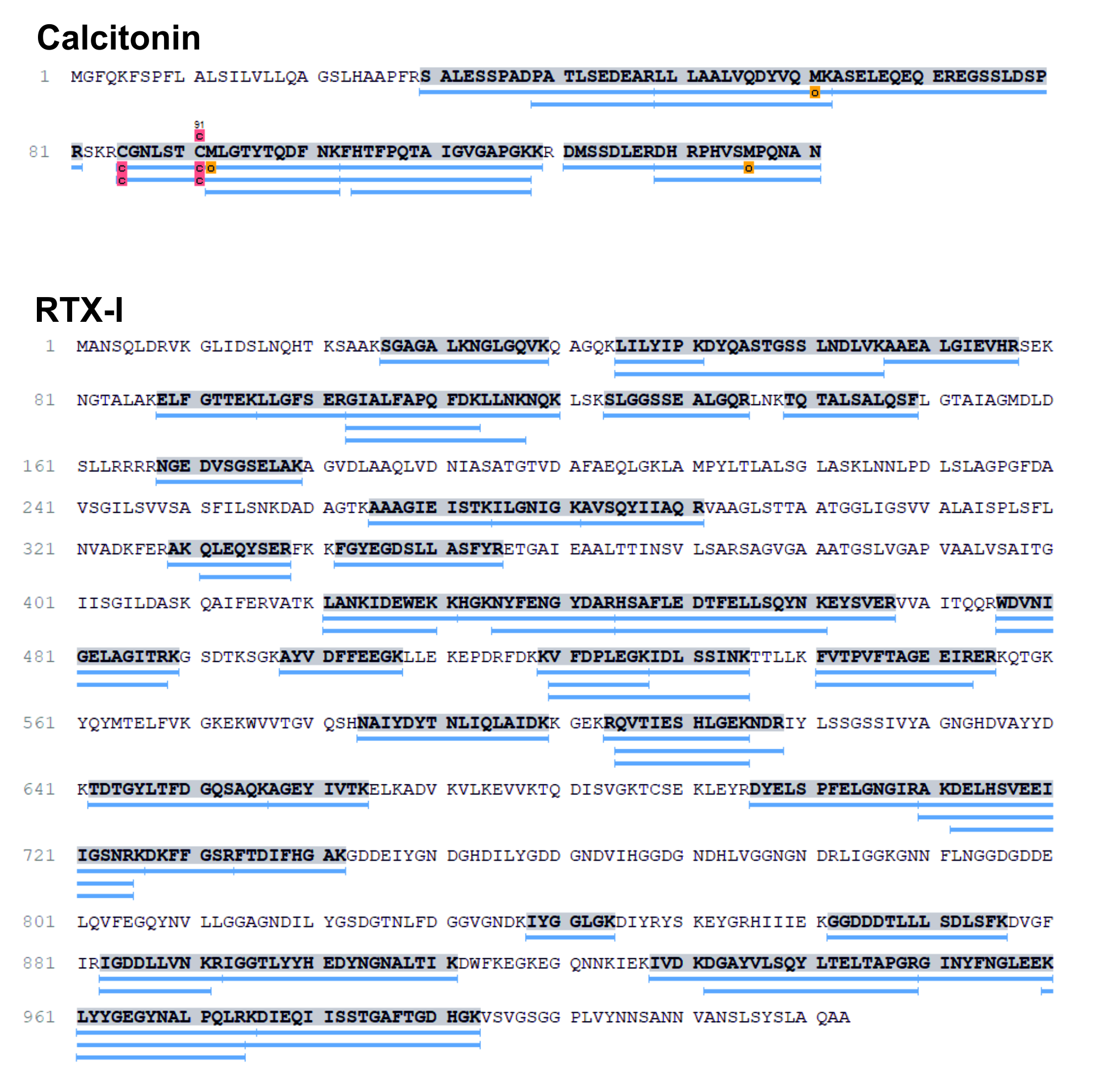

Supplement: Supplementary file 5 — Additional file 5: Figure S5. Sequence coverage of Calcitonin and RTX-I toxin. The blue lines represent identified peptides by LC-MS/MS [file 12014_2023_9410_MOESM5_ESM.tif]
